# High sensitivity of Aeolus UV surface returns to surface reflectivity

**DOI:** 10.1038/s41598-023-44525-5

**Published:** 2023-10-16

**Authors:** L. D. Labzovskii, G. J. van Zadelhoff, L. G. Tilstra, J. de Kloe, D. P. Donovan, A. Stoffelen

**Affiliations:** R&D Satellite and Observations Group, Royal Netherlands Meteorological Institute (KNMI), De Bilt, The Netherlands

**Keywords:** Climate sciences, Atmospheric science

## Abstract

Global ultraviolet (UV) surface reflectivity climatologies are all composed from daylight passive remote sensing observations of reflected UV light, generally integrated over a distribution of attainable reflection directions. We discovered the sensitivity of Aeolus lidar surface returns (LSR) to surface characteristics, providing the first evidence that active remote sensing can be effectively used for retrieving unidirectional UV surface reflectivity on global scales. LSR reproduces surface reflectivity monthly changes in Sahara, which are visible in the Lambertian Equivalent Reflectivity (LER) climatologies from TROPOMI and GOME-2. Very high correlations (r > 0.90) between gridded LSR and the LER climatologies are reported at global and regional scales for 36 different regions. Three clear land cover gradients are discerned from Aeolus LSR signal: (1) water/land, (2) vegetation/arid areas and (3) no snow/snow. The strongest LSR signal was retrieved over snow, while over vegetation, we found moderate negative agreement (r < − 0.60) between LSR and vegetation index proxy. Overall, the success of the first active remote sensing method for retrieving unidirectional UV surface reflectivity using Aeolus is demonstrated. Our approach can be effectively used to detect unresolved land and, especially, snow cover changes in high latitudes because, unlike passive instruments, Aeolus also provided nighttime observations.

## Introduction

The limited knowledge of surface reflectivity characteristics at ultraviolet (UV) wavelengths has been a source of conflicting results in previous studies^1^. This gap is of particular concern since we need to know UV surface reflectance characteristics for understanding human UV exposure and for retrieving accurate aerosol optical depth estimates at 355 nm^2^. Most crucially, the information about surface reflectivity is a prerequisite for any application that requires accurate radiative transfer modelling such as spaceborne remote sensing of trace gases, aerosols/clouds, or chemistry transport models. Besides that, the strong surface reflectance characteristics of white surfaces at the UV wavelength^3,4^ unravel the potential for the detection of snow surfaces or even the classification of snow type (old/new) if an accurate, high-resolution UV surface reflectance map can be generated. This information is essential for avoiding underestimated snow albedo^5^, which can adversely affect passive remote sensing instruments at high latitudes.

Despite the need for accurate surface albedo estimates for climate studies and atmospheric research, our understanding of the global-scale surface reflectivity at the UV spectral band remains limited. To date, all remote sensing studies have relied on passive instruments for retrieving surface reflectivity characteristics in UV, which use the sun and related atmospheric radiation as a source of light for the retrieval. For instance, Lambertian Equivalent Reflectivity (LER) climatologies generated using such methods contain substantial errors, stemming from the necessity to apply atmospheric correction, inflicting more adverse effects at shorter wavelengths^3^. These climatologies do not take into account any observations from high latitude regions during polar night and are based on observations over all solid hemispheric angles, requiring assumptions about Lambertian type of reflectance. Previous studies have indicated that these deficiencies and assumptions can be alleviated by active remote sensing such as lidar sounding^6^, which benefits from the small field of view, the stable; unidirectional source of light from the used laser and the constant viewing angle of the observations. Moreover, lidar observations can provide unidirectional characteristics of surface reflectivity such as attenuated backscatter from surface (sr^−1^)^7^ without assumptions on the heterogeneity and isotropy of the underlying horizontal surface required for passive instruments^1^.

In practice, the earlier work of Reagan and Zelinski^8^ suggested that the lidar sensitivity of white surfaces (e.g., snow) is ~ 20 times greater than that of dark soil at visible wavelengths. Some further studies predicted that the backscatter lidar signals from white surfaces would be especially high at 355 nm^9^. Weiler^4^ more recently used 355 nm data from the Aeolus pre-launch dedicated Airborne Demonstrator consisting of a High-Spectral resolution wind UV lidar that had been specifically developed to follow a similar measurement principle and specification^10^ of the spaceborne instrument ALADIN planned for the Aeolus mission. Most interestingly, the authors^4^ suggested that Lidar Surface Returns (LSR) from fresh snow might be up to ~ 95 times stronger than LSR from grass/soil at UV wavelengths. However, researchers have not yet empirically tested this sensitivity during the in-orbit period of the Aeolus High-Spectral resolution wind UV lidar, which was space-borne from August 2018 until July 2023. The experience with nadir spaceborne lidar systems like CALIOP suggests that the backscattered signal from surface bin can be translated into the BRDF (Bidirectional Reflectance Distribution Function); a function of surface reflectivity characteristics^6^. With the publication of datasets containing calibrated backscatter signals from the Mie channel and surface detection algorithms, the opportunity to explore the sensitivity of lidar to surface reflectance characteristics at 355 nm with the unique optical setup of Aeolus (with the incidence angle of ~ 35°) has arisen as well. Advantageously, the unidirectional sensing allows to probe surface reflectivity at the given angle without necessity to make assumptions on surface properties (e.g., Lambertian) in the reflectivity retrievals. The small ground integration of ~ 3 km of Aeolus also enables more efficient cloud screening due to the inherent sensitivity of Aeolus backscattering signals to clouds^11^.

Our study elucidates the sensitivity of Aeolus LSR (355 nm) to surface reflectance properties to elaborate how this potential opportunity for space-based surface UV reflectivity retrieval can be further exploited. To this end, we used the Lidar Surface Return (LSR) of Aeolus calibrated attenuated backscatter data for the first 12 months of the Aeolus mission (09.2018–08.2019).

## Results

**Figure 1:**
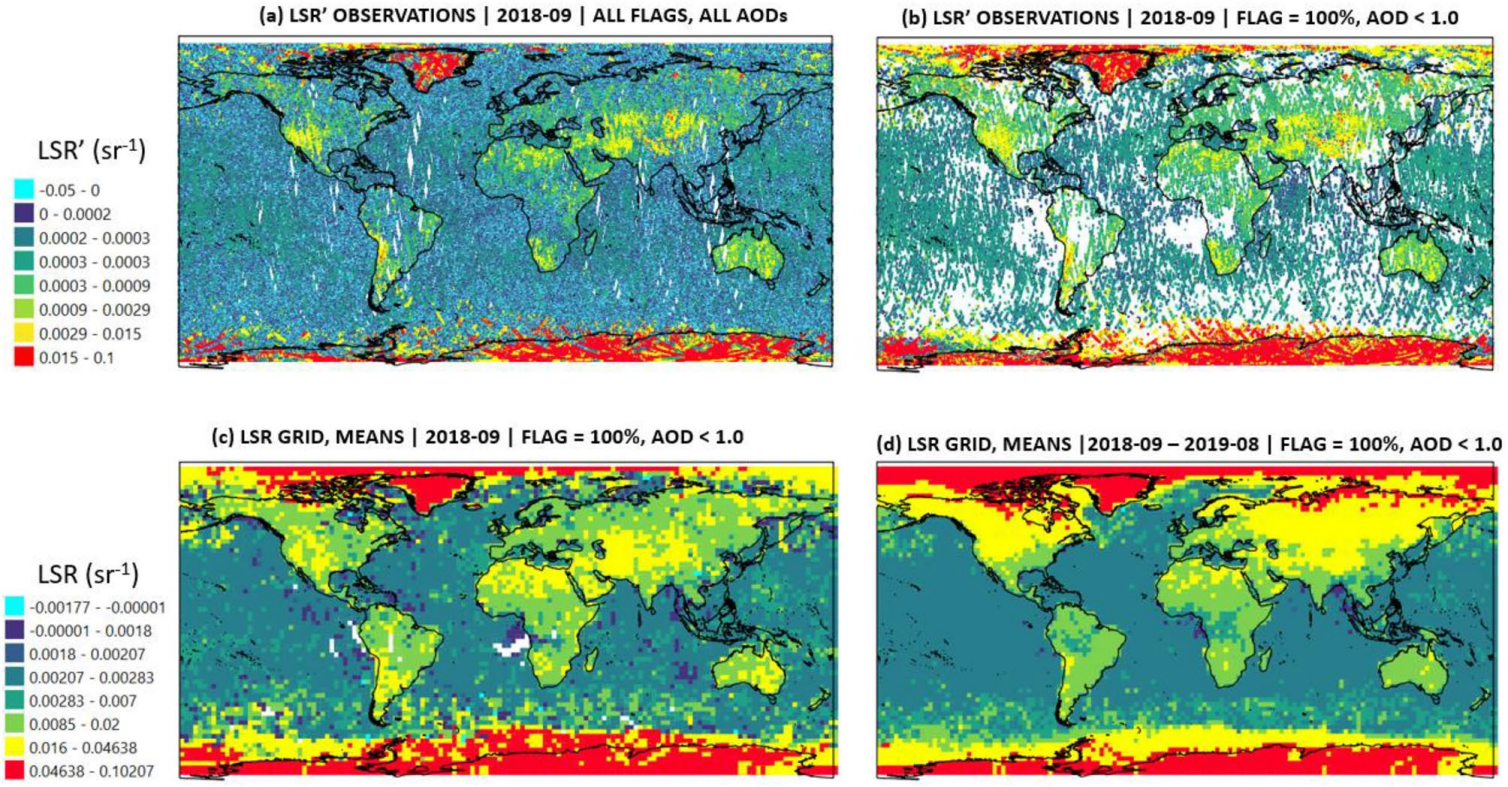
Illustration of the LSR calculation procedure using Aeolus at 355 nm starting from (**a**) uncorrected and unfiltered aggregated monthly observations of LSR’ (sr^−1^) for 2018-09, (**b**) uncorrected, but filtered (Flag = 100%, which means that 100% of attenuated features were filtered out; AOD < 1.0 only) aggregated monthly observations of LSR’; both a and b were calculated using the highest Aeolus measurement resolution. Gridded (2.5° × 2.5°) monthly estimates of corrected LSR for ‘2018-09’ and for the ‘2018-09–2019-08 period’ excluding June 2019 (which is not available) are illustrated in panels (**c**) and (**d**), respectively. This map was created using the open source software QGIS (v 3.22.6) under the GNU General Public License.

In this work, we used the backscatter signal (β) from Aeolus observations to retrieve the surface integrated attenuated backscatter (SIAB) or simply–Lidar Surface Return (LSR) using the experience of previous studies (see section "Methodology" describing methodology for details). In simple sense, LSR represents a UV surface echo, registered at the receiver of Aeolus. We first calculated uncorrected LSRʹ (γʹ in Eq. 1) for every month of the study period. Figure 1a illustrates the monthly aggregated Aeolus orbits for September 2018 including all LSR' observations without any filters applied. In line with previous studies on surface UV reflectivity^4,5^, a simple calculation of LSR' from the surface bin of Aeolus (see section "Methodology" for the definition of the surface bin) for September 2018 yielded the strongest returns from white surfaces (Greenland, Antarctica), followed by surfaces occasionally covered by snow (Tibet, Andes) and arid areas (Sahara, Western U.S., Iran); all visible in Fig. 1a as yellow-colored clusters. As all strong attenuated footprints had to be filtered out, we applied the filtering strategy described in section "Filtering out attenuating features from LSR observations". Figure 1b shows that the filtering removed most of the highly attenuated LSR' estimates (from water and ice clouds), thus resulting in clarified surface reflectance gradients, where the difference between land and water becomes very clear. We also took into account the effects of molecular and aerosol attenuation to ensure that LSR primarily expresses a surface reflection and is clear from atmospheric effects on the signal. As indicated by Fig. 1, the abundance of the data yields a quasi-global LSR coverage. A minor gap near Mid-Western Africa remains due to the high aerosol load in September 2018, driven by the active biomass burning events occurring in September in that region^12^. The same procedure as shown in Fig. 1 (from panel a to c) was applied to every month of the study period. As a result, we obtained a yearly mean of corrected LSR (Fig. 1d). Interestingly, the yearly mean of LSR resembles a surface cover map with four distinct LSR gradients: water–land, dark surface–white surface (snow-covered areas) and perhaps even the vegetation surface–arid surface gradient. A slightly weakened signal over Mid-Western Africa (blue cluster with ~ 0.0018 sr^−1^) is evident. This pattern seemingly points to an artifact from high aerosol load cases, which were partially included in the analysis. Specifically, although we filtered out AOD > 1.0 cases, some fairly high aerosol load cases (AOD 0.75–0.99 for example) were included in the analysis to ensure the abundance of statistics in the analysis. Overall, the clear land cover type-dependent LSR gradients from Fig. 1 confirm that the AOD < 1.0 filtering alleviated attenuation effect while the optimal threshold for filtering out high AOD cases should be a subject of detailed sensitivity analysis.

**Figure 2:**
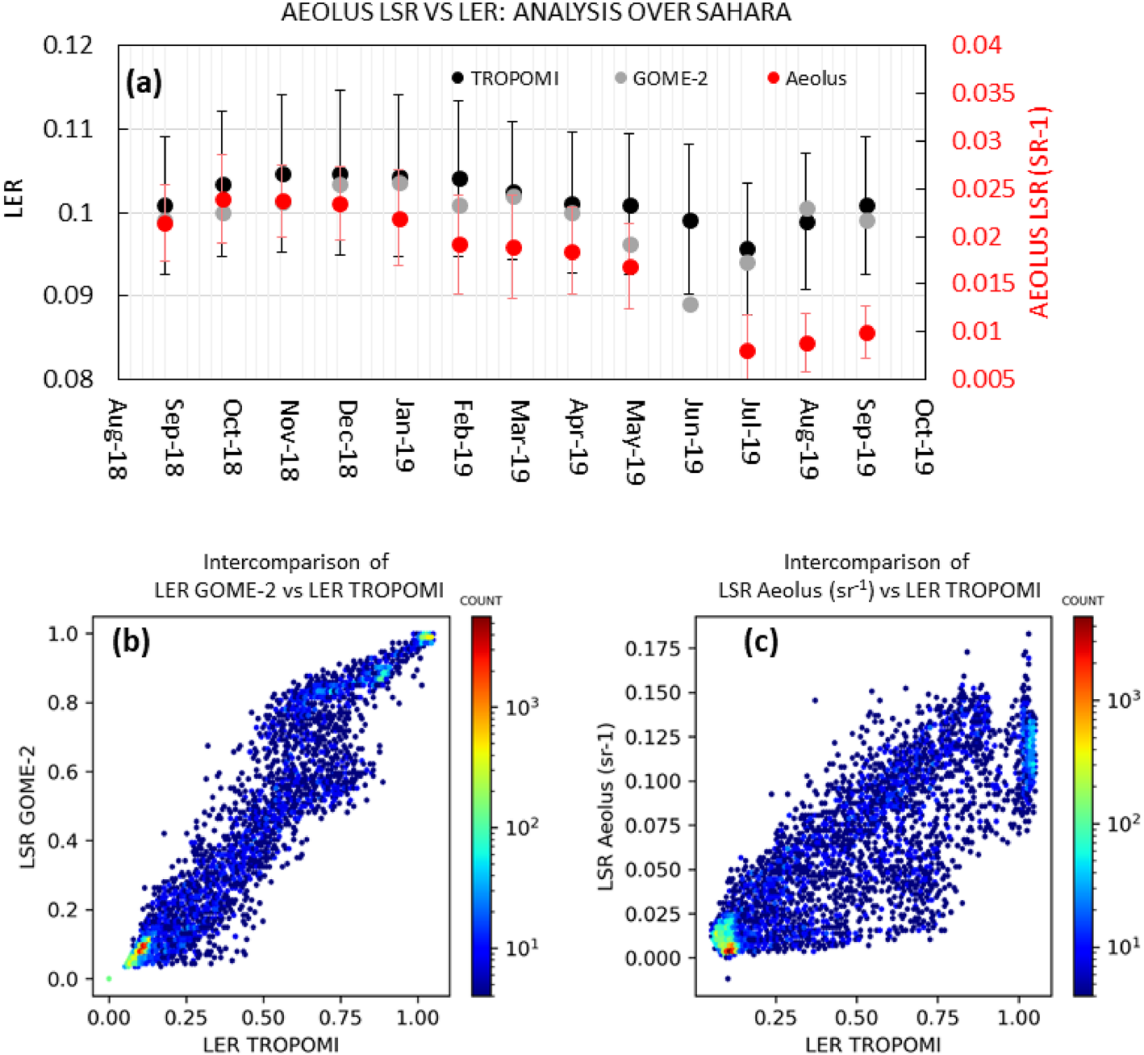
(**a**) Comparison of monthly LSR estimates from Aeolus versus LER estimates for Sahara (uncertainties are taken from the standard deviations of monthly estimates within the region of interest; LER_TRO_ uncertainties are omitted for better visibility of the plot; they are very similar to LER_GOM_ uncertainties). Intercomparison of mean estimates of (**b**) LER_GOM_ vs LER_TRO_, (**c**) LSR Aeolus vs LER_TRO_. All the comparisons were performed at the 2.5° × 2.5° grid resolution for 2018.09–2019.08 in (**b**,**c**), 1 month of additional data (September 2019) was added for (**a**). In (**a**), LER estimates were taken from climatologies, which means that the same LER climatological estimates were used for September 2018 and September 2019. Note that the color bar reflects the number of occurrences plotted in the form of log-normal. Note that Aeolus June 2019 data were not used in this study (see Sect. "Filtering out attenuating features from LSR observations" for the detailed explanation).

We further verify the LSR gradients visible in Fig. 1 and elucidate the sensitivity of LSR to surface reflectance properties at 355 nm. To this end, we calculated the correlation coefficients between LSR estimates and the LER references for 09.2018–08.2019 (Fig. 2) over the Sahara desert and globally. The Sahara was selected as the reference area to evaluate the sensitivity of LSR to surface reflectivity characteristics due to its lowest LER seasonal variability (see SI Fig. S2 in supplement). Interestingly, LSR reproduced even weak surface reflectivity monthly changes in Sahara, which had been previously considered to be negligible in LER (red plot, Fig. 2a). Most importantly, these LSR changes agreed well with LER_TRO_ (correlation coefficient, r = 0.89) and moderately well with LER_GOM_ (r = 0.62) in the study period (09.2018–08.2018). Such agreement can be considered high since the two LER references do not always perfectly agree with each other (r = 0.72 for the Sahara) even over arid regions with the reflectivity characteristics, nearly independent from vegetation changes. Such differences are realistic because the GOME-2 and TROPOMI satellites have different local overpass times, thereby potentially inducing minor differences in the final LER climatologies^13^. Although the surface reflectivity variability is generally weak in the Sahara, the Aeolus LSR not only captures this variability, but also exhibits a wider range of surface reflectance change (0.010–0.031 sr^−1^), compared to LER_TRO_ (0.070–0.120). This observation demonstrates the potential of LSR measurements to detect changes in the surface reflectance. These changes were seemingly captured less clearly by the surface albedo based on passive remote sensing techniques. Due to a noticeable decrease of Aeolus signal (from May to July 2019), we added the September 2019 LSR estimate to the Sahara analysis. Figure 2a clearly shows that the LSR estimate from September 2019 is nearly twice lower than the LER estimate of September 2018. This phenomenon was most likely driven by the switch between two redundant laser transmitters that occurred in June 2019 after deterioration of the signal, which marked the transition between two Flight Modes (FM) of Aeolus: FM-A and FM-B periods^14^. This switch is linked to the change of Aeolus performance that has been thoroughly examined in some previous studies^14,15^. Despite this, the relative sensitivity of LSR to surface reflectivity remained high as the Aeolus LSR is increasing from July 2019 to September 2019. As a result, a good statistical agreement with slowly increasing LER_TRO_ estimates was discerned for this short period. Except the Sahara, we extended our analysis to global scales. As expected, the LER reference climatologies agree very well with each other (r = 0.96) at global scales (Fig. 2b). More interestingly, Aeolus LSR also exhibits very good correlation with LER_TRO_ (r = 0.92) and LER_GOM_ (r = 0.94). Unlike a nearly linear agreement of two different LERs, LSR-LER relationship showed some sigmoid-shape relationship by exhibiting stronger variability in the low return range (0.00–0.01 sr^−1^) and the high return range (0.95–0.99 sr^−1^). We examine this pattern of the LSR variability in detail further.

**Figure 3:**
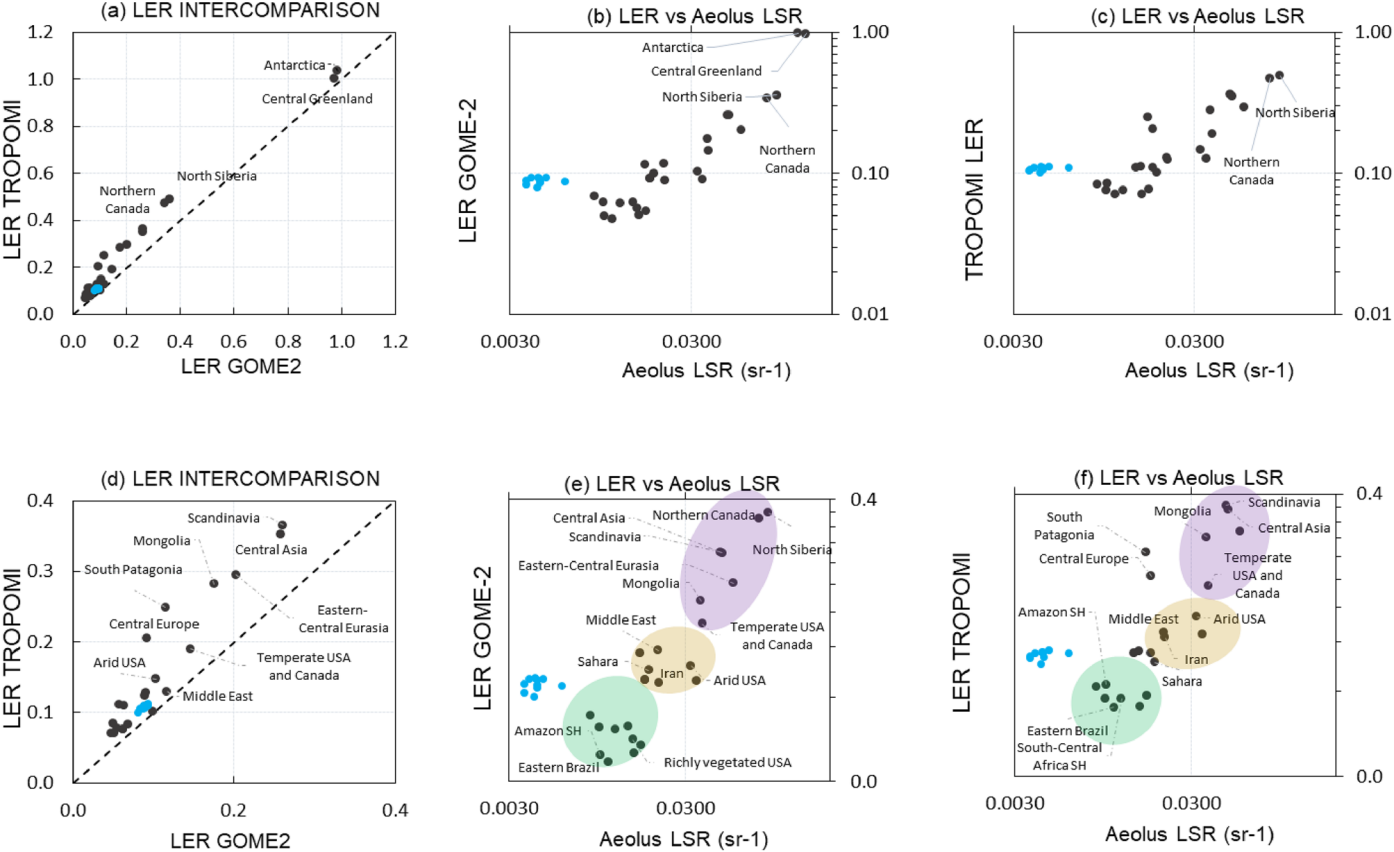
Regional comparison (36 regions) of mean estimates of LSR vs LER_TRO_ and LER_GOM_ for land (black dots) and water (light blue) on 2.5° × 2.5° grid during the study period. Top panels: (**a**) LER intercomparison (linear scales for both axis), (**b**) LER_GOM_ vs LSR Aeolus intercomparison (logarithmic scales for both axis), (**c**) LER_TRO_ vs LSR Aeolus intercomparison (logarithmic scales for both axis). Bottom panel: zoom-in plots into the gray rectangle area shown on top panels for (**d**) LER intercomparison (linear scales for both axis), (**e**) LER_GOM_ vs LSR Aeolus intercomparison (logarithmic scales for both axis), (**f**) LER_TRO_ vs LSR (logarithmic scales for both axis). Colored bubbles represent LSR-based clustering of areas covered by snow during the year (purple), arid areas (yellow) and areas with developed vegetation (green).

To examine the LSR sensitivity to land cover, we calculated the mean LSR estimates for 36 selected regions with distinct land cover characteristics (see the list of this regions in the supplementary material S1) and compared them with mean LER estimates for the same regions (Fig. 3). Figure 3 shows that such coarser, but region-specific analyses yielded nearly perfect correlation between LER_GOM_ and LER_TRO_ (r = 0.99). More interestingly, we found very high correlation between LSR estimates and both LER references with r = 0.94 (LER_TRO_; Fig. 3b) and r = 0.91 (LER_GOM_; Fig. 3c), respectively. The correlation between LSR and LER is driven by land, not water returns. Specifically, the LSR-LER comparison revealed very high correlation between these different parameters over land regions (r = 0.96 for LSR-LER_TRO_ and r = 0.94 for LSR-LER_GOM_, respectively), but very weak correlation (r = 0.47–0.50) over water. This difference is likely attributed to the different physics of Aeolus returns over water. Due to the highly non-nadir observations of Aeolus (incidence angle = 35°), the lidar specular reflection is nearly negligible, compared to nadir systems^16^, resulting in backscatter information mostly from the sub-surface layer of water^17^. This pattern is evident in the LSR-LER comparison as the Aeolus water returns are very weak, while the LER climatology demonstrates relatively stronger reflectivity from water (see blue markers on Fig. 3), which, for instance, exceeds the reflectivity from some tropical forests^3^. Furthermore, this regional analysis can shed light on the sigmoid-shaped pattern of LSR and LSR-LER fundamental differences, illustrated in Fig. 2. Specifically, while the notable sensitivity of both LSR and LER to white surfaces was discerned, Aeolus might be more sensitive to some changes in surface reflectance that have been previously considered below the measurement ability of passive remote sensing systems. To illustrate this effect, Fig. 3 shows both full-scale and zoomed-in ranges of Aeolus LSR in logarithmic scales (see top and bottom panels, respectively). As seen from Fig. 3e,f, the mean LSR estimates clearly change depending on the land cover type. LSR increases from water (blue dots) to vegetated areas (green cluster), to arid zones (yellow cluster) with the highest resultant returns from snow/ice-affected zones (purple cluster). This finding agrees with some previous works, such as Chadysiene and Girgzdys^9^ who had demonstrated that snow and sand surfaces reflect ~ 80% and ~ 25% of UV radiation, respectively, while grass yields the weakest reflectance. More interestingly, the difference between the strongest signal (snow) and weakest signal (water) in LSR, we revealed, is similar to the difference between dark and white surfaces, reported by the early work of Reagan and Zielinski^8^. The LSR range in the zoomed-in analysis unveils three evident clusters of LSR in ascending order of signal strength: vegetated surface (green bubble), arid surface (yellow bubble) and snow surface (purple bubble). The differences in the shapes of the clusters between the Aeolus-GOME-2 and Aeolus-TROPOMI plots can be attributed to the different effects of snow/ice surfaces, captured in the LER_GOM_ and LER_TRO_ products based on the different instruments and the different observation years (see ‘bubbles’ in Fig. 3e,f). To clarify this aspect, we examined the sensitivity of Aeolus LSR to the change of reflectance characteristics of the surface depending on vegetation using a simple correlation analysis against NDVI. Indicatively, neither LER_TRO_, nor LER_GOM_ exhibited any association with mean monthly NDVI estimates for the same period and same regions as in Fig. 3, but LSR from Aeolus exhibited a moderate negative correlation (r = − 0.61). This result suggests a potential sensitivity of Aeolus signal to vegetation changes. In particular, the strongest returns come from the regions with considerable snow cover (low, but not negative NDVI like for homogeneously white surfaces) and dramatically weaken with denser vegetation (from NDVI = 0.1–0.3 for arid zones to NDVI > 0.6 for various productive ecosystems).

## Discussion and conclusions

The sensitivity of Aeolus Lidar Surface Returns (LSR) to the land surface reflectivity, land cover type and even to vegetation changes was demonstrated for the first year of the Aeolus flight (09.2018–08.2019). The very good statistical agreements between LSR and the existing LER climatologies at local, regional and global scales; all clearly indicate that LSR can be used to express the unidirectional UV surface reflectivity. Unlike passive remote sensing-based approaches to retrieve surface reflectivity, Aeolus LSR does not require any assumptions on the irradiation at the surface at all angles and the surface properties (isotropy, heterogeneity), thus widening the spatial coverage of surface UV reflectivity observations to high latitudes and extending the observational coverage to the global scales. Besides that, the consistent difference between LSR (unidirectional) and LER (with assumptions on daylight and surface properties) can be used to understand how to convert LSR to LER at UV and highly non-nadir incidence, which is currently rather unclear. In particular, should one divide the hemispheric solid angle of 2π^6^ or apply the theoretical considerations of Josset et. al^17^ with more complex parametrization of reflection and multiple scattering. In this way, LSR can be linked to the surface reflectivity quantities, used in radiative transfer modelling, such as surface albedo, and better relate to LER from passive instruments in future studies. Aeolus LSR can be advantageously used to confirm or refute such assumptions, because it is better resolved in both illumination and reflection angle. Overall, given the sensitivity of LSR to surface reflectivity and land type, LSR product can be potentially incorporated into the official L2A Aeolus data. From the scientific perspective, such dataset can be already applied for (a) snow detection/snow cover change detection studies, especially if such change is related to black carbon deposition^18^, which dramatically weakens albedo of snow according to laboratory experiments^19^. For future missions with lidars on-board^20^, like EarthCARE and Aeolus-2, the accurate information about LSR can be utilized (b) as the input in the lidar equation for better constraining the forward modelling of the main lidar equation and (c) for calibrating the lidar instruments used in such missions^21^ by utilizing UV reflectance information from strongly reflecting white surfaces. Lastly, (d) considering the abundance of Aeolus observations, LSR can be used to improve land cover classification accuracy by applying deep learning models for determining the range of LSR characteristic for each type of land cover.

## Methodology

### Surface detection and LSR retrieval

Launched in 2018, Aeolus carries the Atmospheric Laser Doppler Instrument (ALADIN), a high-spectral resolution UV lidar (355 nm) with an incidence angle of ~ 35° and the diode-pumped frequency-tripled Nd:YAG laser within. The instrument provides information on the lowest ~ 30 km of the atmosphere for 15.6 orbits per day with a 06:00 and 18:00 local solar time (LST) Equator overpass in a sun-synchronous orbit and with a 7 day repeat cycle. ALADIN emits pulses of 80 mJ and has a 50 Hz pulse repetition frequency. It is equipped with both Rayleigh and Mie channels, which allows to retrieve backscattering signals from molecules and aerosols, respectively. The Aeolus laser has a field of view of 19 μrad resulting in a footprint is ~ 12–15 m. The highest horizontal resolution of the Aeolus measurements is ~ 3 km. The Aeolus measurements are accumulated on board to decrease the read-out noise in the downlinked data. To ensure enough signal is available for the ground processing a number of 19 pulses were accumulated in the first years of the mission (our study period falls within this period). This accumulated result is the highest resolution data available for users, referred to as “measurement level” and was used in this study for analysis. The Aeolus profiles each consist of 24 bins with the vertical range bin size varying from 250 to 2000 m depending on the height, while the range bin settings (as the height of the lowest bin for instance) can vary depending on the time and location of Aeolus measurement^10^.

As mentioned in the results, we used the backscatter signal (β) from ALADIN as a measure of the amount of light that is reflected back to the lidar receiver from the atmosphere/ground in order to retrieve the surface integrated attenuated backscatter (SIAB) or simply–Lidar Surface Return (LSR). This procedure is based on the experience of previous CALIPSO-based lidar studies focused on lidar surface signal^6,7^ Eq. (1) calculates the uncorrected LSR (γʹ, sr^−1^) by multiplying the surface attenuated backscatter (β_surf_, sr^−1^ m^−1^) determined by the closest range gate to the surface in Digital Elevation Model (DEM) on the surface range bin width of Aeolus (Δr_surf_, m). Note that surface attenuated backscatter is taken from Aeolus L2 data described in section "Data" below. DEM is the modelled estimate based on the available knowledge about the satellite position and laser pointing geometry used from the ACE V2 dataset (300 m × 300 m, 9 arcsecond resolution). Since Aeolus range bins are very large (> 250 m), we consider only the bin that has the closest intersection with the DEM. Next, we corrected molecular and aerosol contributions using Eq. (2) and obtained final surface-based LSR estimates (γ). Eq. (2) shows how molecular and aerosol corrections were applied using the Rayleigh optical depth (OD_Ray_; see Supplementary Material, S1) and Aerosol Optical Depth (AOD; see Supplementary Material, S2), respectively. In theory, LSR over land can be converted into BRDF using a 2π correction factor, while such conversion has been applied mostly to the nadir looking CALIOP with the incidence angle close to 3°^6^. The same approach might not be applicable for highly non-nadir lidars like Aeolus because one does not take into account the angles of incidence and refraction. Over water surfaces where much more complex interaction between specular, whitecap and subsurface reflectance components may occur the LSR and BRDF comparison is more complicated^17^.

1 $$\gamma {\prime}={\beta }_{surf}\times \Delta {r}_{surf}$$

2 $$\gamma = \gamma \mathrm{^{\prime}}{e}^{2(AOD+{OD}_{Ray})}$$

### Filtering out attenuating features from LSR observations

To ensure that LSR is not attenuated by high AOD conditions and cirrus clouds, we exclude all LSR measurements with AOD > 1.0 We calculated AOD using the Aeolus Profile Processor Algorithm (AEL-PRO), which relies on the optimal estimation and forward modelling inversion procedure. In short, AEL_PRO retrieves the lidar-to-backscattering ratio profile by using only the pure Rayleigh and Mie attenuated backscatter values as input, thereby yielding accurate extinction coefficient profiles^22^. The output profiles of the retrieved state vector, including aerosol/cloud extinction coefficients, were utilized in this work to estimate AOD. Moreover, since AEL_PRO can categorize atmospheric features (water-cloud, ice-cloud, aerosol, clean sky, etc.), we applied the most stringent filtering strategy by excluding any LSR observations potentially contaminated by ice cloud and water cloud presence. In simple words, we used AEL_PRO to keep only the high quality LSR observations without clouds, where the surface signal was not attenuated. This filtering was performed at the highest measurement resolution of Aeolus. For AEL_PRO details, see supplementary material S2 and Donovan et al.^22^ work. We subsequently calculated monthly averages of the LSR with the corresponding standard deviations on a 2.5° × 2.5° grid in the first full yearly (or seasonal) cycle of Aeolus observations (September 2018–August 2019). In the study period, the monthly averaging of millions of observations yields an abundant quasi-global coverage by LSR observations. Minor temporal data gaps were present only during some days in January and February 2019, when Aeolus experienced a system failure. We also did not use any data from June 2019 due to the change from the Flight model-A laser, FM-A, to the second laser, FM-B, period^14^ to avoid any negative effects of the shift of the regime during the same month. Each step of the LSR calculation is illustrated in detail in the supplementary material (SI Fig. S1). Note that although we thoroughly addressed all potentially malignant effects for LSR estimation, some limitations stem directly from the Aeolus setup. Most importantly, the emitted lidar pulse is circularly polarized; however, the Aeolus receiver is only detecting the co-polar component, which could lead to discrepancies in the LSR estimations. This limitation is inherent as Aeolus does not have a depolarization channel. Future LSR estimations from Aeolus may be revisited when the EarthCARE mission is launched, which includes a linearly polarized lidar instrument at the same wavelength with a depolarization channel. This will allow an estimation of both the circular and co-polar components of depolarization, which can then be compared to the Aeolus LSR estimates in a retrospective analysis.

### Data

This study primarily relies on Aeolus data including the Level 1B (L1B) reprocessing product^22,23^ for detecting the surface at the highest spatio-temporal resolution of Aeolus sounding. The L1B product provides geolocation, DEM, and land/water type classification information. The L2A AEL_PRO dataset contains cross-talk-corrected observations, providing the attenuated particle backscattering (for the LSR calculation), the retrieved extinction coefficient profile (for AOD filtering of high aerosol/cloud load cases and for correction of LSR for weak aerosol/cloud attenuation), the attenuated molecular backscattering (for OD_Ray_ correction and molecular contribution), and associated uncertainties. Note that cross-talk represents a substantial source of noise present in both the Mie and Rayleigh channels. In simple words, it is contamination of either of the channels by the signal from the other channel. Cross-talk is an adverse feature for determining backscattering ratio and is therefore detrimental for lidar-based estimation of atmospheric backscattering and extinction profiles. See the details on the AEL_PRO data and cross-talk correction in the supplementary material (Sect. S2). For validation, we used Lambertian Equivalent Reflectivity (LER) estimates from TROPOMI (TROPOspheric Monitoring Instrument) and GOME-2 (The Global Ozone Monitoring Experiment-2 (GOME-2), referred to as LER_TRO_ and LER_GOM_, respectively. Note that LER is derived from the specific solar geometry sampled, whereas the satellite viewing dependence is removed by assuming a Lambertian surface. LER expresses the omnidirectional surface reflectance characteristics, though not all surfaces may be Lambertian. Due to the inherent LSR-LER differences and lack of clear pathway how to translate LSR to BRDF at 35°^17^, we did not apply the normalization and physical unit conversion but evaluated statistical agreement between LSR and LER. We accessed TROPOMI (minimum LER with snow/ice v1.1) and GOME-2 LER (mode LER, v4.0) at 354 nm (the closest wavelength to the 355 nm wavelength of Aeolus) monthly climatologies^3,24^ with the highest spatial resolution available of 0.125° × 0.125° and 0.25° × 0.25°, respectively. They were resampled to 2.5° × 2.5° grids with the uncertainties as the standard deviation of LER during the month. LER was downloaded from the TEMIS (Tropospheric Emission Monitoring Internet Service) website (https://www.temis.nl/surface/albedo/, accessed on 12.02.2023). We used LER estimates, reflecting snow-affected areas as well because it is crucial to include snow/ice regions in the analysis to evaluate the sensitivity of Aeolus LSR to the strongest white reflectors. Note that some differences between LER_TRO_ and LER_GOM_ are plausible: LER_GOM_ relies on a statistical filtering, while LER_TRO_ relies on active cloud filtering. The overpass times of the satellites are not the same, which means that the sun is in a different position and the presence of surface BRDF-related effects or atmospheric effects can enhance the LER differences. Presumably, there is a dependence of atmospheric accuracy on the sun angle as well. We also used the difference between the near-infrared and red light reflected from the surface divided by their sum, i.e., the Normalized Difference Vegetation Index (NDVI) estimates from MOD13C2, v006, which reflect the vegetation conditions (mostly extent and productivity) of a given area. We accessed these estimates from the GIOVANNI open database (identifier: 10.5067/MODIS/MOD13C2.006, accessed from https://giovanni.gsfc.nasa.gov on 01.11.2022) with 0.05° resolution downscaled to 0.25° × 0.25° and averaged (per month).

### Supplementary Information

Supplementary Information.

## Data Availability

The data we used is available through the following sources. The analysis in this paper is based on Aeolus Level 1B products; this data is publicly available and can be accessed via the ESA Aeolus Online Dissemination System: https://aeolus-ds.eo.esa.int/oads/access/. The L1B products are processed in the framework of the second and third Aeolus reprocessing campaigns. Furthermore, TROPOMI and GOME-2 LER data is available at https://www.temis.nl/surface/albedo/tropomi_ler.php according to Tilstra et al.^24^, MODIS NDVI data is available at https://giovanni.gsfc.nasa.gov (MOD13C2, v006) according to 10.5067/MODIS/MOD13C2.006.
