# Supplementary material for: High sensitivity of Aeolus UV surface returns to surface reflectivity
Source: Sci Rep. 2023 Oct 16;13:17552. doi: 10.1038/s41598-023-44525-5 (PMC10579410; doi:10.1038/s41598-023-44525-5)
Supplement: Supplementary file 1 — Supplementary Information. [file 41598_2023_44525_MOESM1_ESM.docx]

SUPPLEMENTARY MATERIAL

S.1 Rayleigh Optical Depth (OD_Ray_) calculation

Since the Aeolus lidar (ALADIN) has a small field-of-view (Lux et al., 2018; Reitebuch et al. 2018) in order to correct the surface return for the effects of attenuation due to Rayleigh scattering it is sufficient to use Beer’s law (i.e. single-scattering). To do this, the Rayleigh scattering optical depth between the surface and the lidar must be calculated.

In order to calculate the Rayleigh optical, we obtained the Rayleigh extinction coefficient ($\alpha_{m}$) profiles from the Aeolus L2A data stream. These values are determined from the atmospheric density profile (ultimately derived from ECMWF forecast data). Eq (S-1) below describes the calculation of OD_Ray_ by integrating Rayleigh extinction coefficient between the surface and the Aeolus top altitude and accounting for the effects of partially filled surface bin and “missed” optical depth above the Aeolus top-bin

| $OD_{Ray}=\sum_{i_{s}}^{i_{t}} \alpha_{m}\left( z_{i} \right)\Delta r\left( z_{i} \right)-k1+k2$ | (S-1) |
| --- | --- |

where $z$ is the altitude, $i\_t$ is the top range index, $i\_s$ is the surface range index and Δr is the range-bin-thickness taking into account the Aeolus pointing angle. k_1_ and k_2_ represent two correction factors that need to be considered. The Aeolus range-bins can be on the order of a few hundred meters even near the surface. Accordingly, we have introduced the k_1_ factor which accounts for the potential over estimation in (S-1) due to the surface being above the lower boundary of the surface-bin. This factor is calculated using the molecular extinction coefficient at the surface bin and the difference in the top boundary of the surface bin and the expected surface height according to the Digital Elevation Map (DEM) information included in the Aeolus products used here.

The correction factor k_2_ is required to alleviate the difference between the changing top bin of Aeolus range gate and the top of the atmosphere. In other words, we need to account the whole atmospheric OD_Ray_, but different portions of Rayleigh signal would be overlooked depending on the location of Aeolus. To this end, k_2_ is introduced and is calculated by Eq. S-2 (Stephens, 1994) considering the pressure at the top of the Aeolus profile (p_t_ [mb]) and the highest range gate altitude (z_t_) [km] and the Aeolus wavelength of 355 nm (λ).

| $k_{2}=\mu^{-1}\frac{p_{t}}{1013.25} 0.0008 \lambda^{\left( -4.15+0.2 \lambda\right) {}}e^{\left( -0.1188z_{t}-0.0016z_{t}^{2} \right)}$ | (S-2) |
| --- | --- |

where μ is the cosine of the Aeolus off-nadir pointing angle (usually ~35^o^)

**S.2 Aerosol optical depth**

In addition to molecular attenuation, the effects of Aerosol (and thin cloud) attenuation must be accounted for in order to estimate accurate surface returns (e.g., LSR from the main text). Here, we have used the AOD corresponding to the aerosol and thin cloud extinction profiles retrieved by the so-called Aeolus Profile Processor algorithm (AEL-PRO). AEL-PRO is an optimal estimation (Rodgers 2000) forward-modelling inversion procedure. Only a brief overview is given here, and more detail may be found in Donovan (2021). Since ALADIN is a type of High-Spectral Resolution lidar (HSRL), the pure Rayleigh and Mie attenuated backscatters can be estimated. The ability to do this means that the extinction profile can be retrieved without assuming the lidar extinction-to-backscatter ratio profile (Shipley 1983) which, in principle, yields more accurate extinction coefficients compared to elastic lidar based techniques. AEL-PRO uses both the pure Rayleigh and Mie attenuated backscatters as input.

As with any optimal-estimation approach, a cost-function is formed which that characterizes the likelihood of the measurements being what they were given a particular parameter configuration of an appropriate forward model combined with our expectations concerning the forward-model parameters. The desired solution is then obtained by numerically minimizing this function.

The AEL-PRO cost function can be written as:

| $\chi^{2}=\left[ \mathbf{y}\mathbf{-}\mathbf{F}\left( \mathbf{x} \right) \right]^{\boldsymbol{T}}\mathbf{S}_{\mathbf{e}}^{\mathbf{-}\mathbf{1}}\left[ \mathbf{y}\mathbf{-}\mathbf{F}\left( \mathbf{x} \right) \right]\mathbf{+}\left[ \mathbf{x}\mathbf{-}\mathbf{x}_{\mathbf{a}} \right]^{\mathbf{T}}\mathbf{S}_{\mathbf{a}}^{\mathbf{-}\mathbf{1}}\left[ \mathbf{x}\mathbf{-}\mathbf{x}_{\mathbf{a}} \right]\boldsymbol{.}$ |  |
| --- | --- |

where the *state-vector* (**x**) is comprised of the log of the per-range-gate extinction coefficients, the corresponding extinction-to-backscatter ratios (S), particle effective radii (Ra) and effective lidar calibration coefficient (C_lid_) i.e

$\mathbf{x}=log_{10}\left[ \left( \alpha_{1},\alpha_{2},\ldots,\alpha_{n},S_{1},S_{2},\ldots,S_{n},Ra_{1},Ra_{2},\ldots,Ra_{n},C_{lid} \right) \right]^{T}$.

$\mathbf{F}\left( \mathbf{x} \right)$ is the forward-model vector which is used to model the observations given the state-vector as input. Here a form of the lidar equation that can approximately account for multiple-scattering (using the effective particle sizes) is used.

The *observation-vector* (**y**) is formed from the observed crosstalk corrected Rayleigh (B_R_) and Mie (B_M_) attenuated backscatters i.e.

$\mathbf{y}\boldsymbol{=}\left( B_{R,1},B_{R,2},..B_{R,n},B_{M,1},B_{M,2},\ldots.B_{M,n} \right)^{T}$.

$\mathbf{x}_{\boldsymbol{a}}$ is the logarithmic a-priori state vector. Here defined as a vector consisting of the log base 10 values of the a-priori lidar-ratios, effective area particle sizes and the value of C_lid_ appropriate for calibrated attenuated backscatter signals (i.e. 1.0).

|  | $\mathbf{x}_{\mathbf{a}}=\mathrm{lo}g_{10}\left[ \left( S_{a,1},S_{a,2},\ldots,S_{a,n},Ra_{a,1},Ra_{a,2},\ldots,Ra_{a,n},1 \right) \right]^{T}.$ |  |
| --- | --- | --- |

$\mathbf{S}_{\mathbf{e}}$ is the observation error covariance matrix and $\mathbf{S}_{\mathbf{a}}$ is the a priori error covariance matrix appropriate to the logarithmic state-vector.

AEL-PRO outputs profiles of the retrieved state-vector including the aerosol/cloud extinction coefficients that are used to estimate the AOD in this work. A simple categorization (e.g. water-cloud, ice-cloud or aerosol) based on thresholds in the retrieved backscatter profiles together with the atmospheric temperature profile is also provided.

**S.3 From uncorrected lidar surface returns (LSR’) to monthly averages of Rayleigh and Aerosol-corrected LSR estimates at 2.5^o^ x 2.5^o^ grid**

Here, we shortly illustrate our methodology for calculating final monthly averages from LSR. SI Figure 1 shows the entire processing routine of LSR, whereas blue blocks represent the steps of processing and red blocks show the LSR results as output of the upper processing step (in chronological order from 1 to 3). As described in the methodology, at first we detected the surface bin of Aeolus using DEM estimate as a reference. For this surface bin, we calculated LSR’ (uncorrected) for all available observations (masks/filtered not applied). As a result of this step, we obtained millions of LSR’ estimates and classified them depending on the month and year of observations within the study period (08.2018 - 09.2019) (see block 1 in SI Figure 1). Then, we applied filtering of these observations using AEL_FM and AOD AEL_PRO products using the following criteria: QFLAG = 100% (only clear, thin cloud and thin aerosol scenes are included in the analysis) and AOD < 1.0, respectively. As a result, we obtained uncorrected LSR’ estimates from “clean” observations only, thereby minimizing the possibility of signal attenuation (block 2 in SI Figure 1). Next, we performed two steps including: calculation of LSR by correcting AOD and OD_Ray_ contributions from LSR’ and calculation of mean estimates of LSR (alongside standard deviations, reflecting systematic uncertainties) at 2.5 x 2.5^o^ grid cell resolution. The output of monthly mean LSR estimates are shown on block 3 (SI Figure 1). To calculate yearly mean LSR estimates (or quasi-yearly mean considering the absence of June 2019 data), we calculated means and standard deviations of monthly averaged LSR in 09.2018 – 08.2019 at 2.5 x 2.5^o^ resolution (see block 4, SI Figure 1).


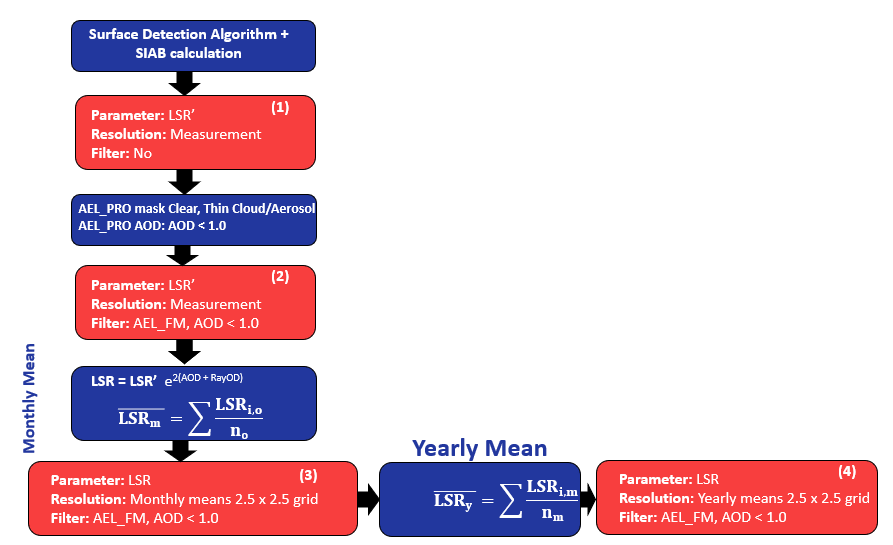


SI Figure 1 Calculation of LSR, processing chain in chronological order from 1 to 4. Blue blocks represent the processing masks/filters, red blocks represent the output of LSR estimates at different stages of processing. All acronyms and terms are defined in the main text of the manuscript, subscripts n, o, m, y denote number of samples, observation resolution, monthly resolution and yearly resolution, respectively.

**S.4 Additional results and information**

SI Figure 2 below illustrates the variability of LER_TRO_ at global scales at 2.5 x 2.5 grid cell resolution. As seen from this figure, the lowest LER_TRO_ variability was revealed in Sahara and in Australia (< 0.005). Note that the variability was calculated using standard deviation of LER across all the monthly means (January – December), available in TROPOMI LER climatology.


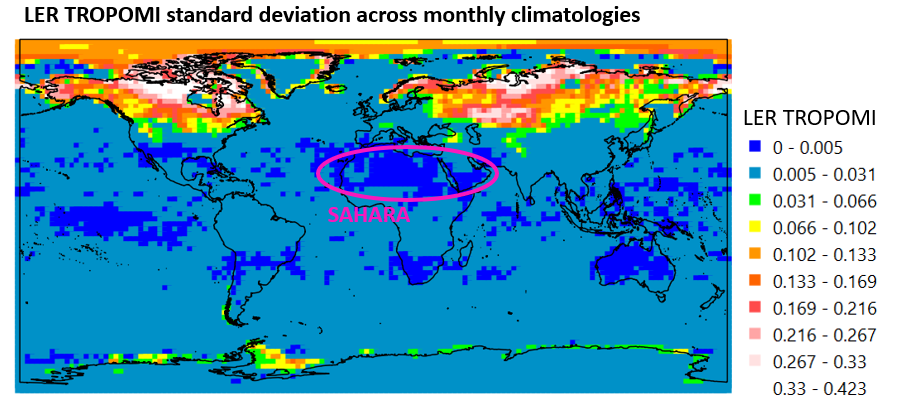


SI Figure 2. Standard deviation of LER_TRO_ based on yearly climatologies (January – December). This map was created using the open source software QGIS (v 3.22.6) under the GNU General Public License.

SI Table 1 below lists all the regions used in the regional analysis in the manuscript (Figure 3).

SI Table 1 list of regions used in the analysis for Figure 3.

| Region |
| --- |
| Amazon SH |
| Antarctica |
| Arid USA |
| Australia |
| Center-Eastern China |
| Central Asia |
| Central Europe |
| Central Greenland |
| Eastern Brazil |
| Eastern-Central Eurasia |
| India-Bangladesh-Nepal |
| Indochinese Peninsula |
| Iran |
| Mexico |
| Middle East |
| Mongolia |
| North Siberia |
| Northern Canada (Areas with abundant snow cover) |
| Richly vegetated USA |
| Sahara |
| Scandinavia |
| South Patagonia |
| South-Central Africa SH |
| Sub Sahel Africa with vegetation |
| Temperate USA and Canada |
| Tibet |
| Caribbean Sea |
| Guinea Gulf |
| North Atlantic |
| North Indian Ocean |
| North Pacific |
| Oceania Waters |
| Philippine Sea |
| South Indian Ocean |
| South Pacific |
| Tasman Sea |

Donovan, D., van Zadelhoff, G.-J., Wang, P., and Huber, D.: ATILD cloud/aerosol algorithms applied to ALADIN, EGU General Assembly 2021, online, 19–30 Apr 2021, EGU21-15189, https://doi.org/10.5194/egusphere-egu21-15189, 2021.

Lux, S., Reitebuch, O., Huber, D., Nikolaus, I. (2018). ADM-Aeolus Algorithm Theoretical Basis Document ATBD Level1B Products. 45. https://earth.esa.int/eogateway/documents/20142/37627/Aeolus-L1B-Algorithm-ATBD.pdf (last accessed: 23 February 2023).

Reitebuch, O., Huber, D., Nikolaus, I. (2018a). ADM-Aeolus Algorithm Theoretical Basis Document ATBD Level1B Products. 45. https://earth.esa.int/eogateway/documents/20142/37627/Aeolus-L1B-Algorithm-ATBD.pdf (last accessed: 23 February 2023).

Rodgers, 2000 C.D. Rodgers Inverse Methods for Atmospheric Sounding

WORLD SCIENTIFIC (2000), 10.1142/3171, URL https://www.worldscientific.com/doi/abs/10.1142/3171

Shipley, S.T., D.H. Tracy, E.W. Eloranta, J.T. Trauger, J.T. Sroga, F.L. Roesler and J.A. Weinman, "A High Spectral Resolution Lidar to measure optical scattering properties of atmospheric aerosols , Part I: Instrumentation and theory" Applied Optics, 23, 3716-3724, 1983.

Stephens, G. L., 1994: Remote Sensing of the Lower Atmosphere.

Oxford University Press, 523 pps
